# Comparison of psychological interventions for anxiety, depression, fatigue and quality of life in colorectal cancer survivors: A systematic review and network meta-analysis protocol

**DOI:** 10.1371/journal.pone.0298589

**Published:** 2024-04-01

**Authors:** Yujie Liang, Xu Zhang, Shan Li, Zhiwen Wang

**Affiliations:** 1 School of Nursing, Jilin Medical University, Jilin, China; 2 School of Nursing, Peking University, Haidian District, Beijing, China; 3 Xinjiang Second Medical College, Karamay, China; 4 Peking University Health Science Centre for Evidence Based Nursing a Joanna Briggs Institute Affiliated Group, Haidian District, Beijing, China; University of Toronto, CANADA

## Abstract

**Background:**

Previous studies have found that psychological interventions have a positive effect on improving physical and psychological problems in colorectal cancer survivors. However, there is still a lack of high-quality evidence reviews that summarize and compare the impact of different psychological interventions. The aim of this study was to synthesize existing psychological interventions and use network meta-analysis to explore whether psychological interventions improve anxiety, depression, fatigue and quality of life in colorectal cancer (CRC) survivors.

**Methods:**

We will extract relevant randomized controlled trials of psychological interventions for CRC survivors from eight electronic databases, including PubMed, Embase, The Cochrane Library, Web of Science, CINAHL, PsycInFO, CNKI, and Wanfang database. Two reviewers will independently screen the literature and extract data. The risk of bias of the included studies will be assessed using the RoB2: Revised Cochrane Risk of Bias Tool. We will then conduct paired meta-analyses and network meta-analyses of the extracted data, using a frequency-based framework and random effects models.

**Discussion:**

To the best of our knowledge, this study is the first proposed qualitative and quantitative integration of existing evidence using systematic evaluation and network meta-analysis. This study will inform health policy makers, healthcare providers’ clinical intervention choices and guideline revisions, and will help to reduce depression and anxiety in CRC survivors, reduce fatigue, improve quality of life.

## Introduction

In 2020, the number of new cases of colorectal cancer (CRC) in the world was 1.88 million, accounting for about 9.7% of all malignant neoplasms, and it is the third most common malignant neoplasm; the number of deaths reached 916,000, accounting for about 9.2% of all malignant neoplasm deaths, and it is the 2nd most common cause of death of malignant neoplasms [1]. Similar to the trend of cancer epidemiology in the world, CRC ranks 2nd in the number of incidence and 4th in the number of deaths among all malignant tumors in China [2]. Fortunately, with the advancement of diagnostic methods, the emergence of precision therapies such as targeted and immunotherapeutic drugs, as well as standardized and orderly diagnosis, staging, and multidisciplinary treatment based on the clinical behaviors of CRC, the survival rate of CRC has been significantly improved [3]. However, in the face of the threat of cancer, adjuvant therapy, and changes in elimination patterns, survivors with CRC experience many psychological problems that require the attention of healthcare professionals [4].

Anxiety and depression are common psychological problems among CRC survivors, with prevalence rates ranging from 1.6 to 57% for depression and 1.0 to 47.2% for anxiety [4]. One cohort study found that each 1 standard deviation increase in anxiety or depressive symptoms was associated with a 16% increase in the risk of death, and that greater anxiety and depressive symptoms not only impede adherence to healthy habits and reduce quality of life in cancer patients, but may also be a marker of accelerated CRC progression [5]. Anxiety and depression are also thought to be related to factors such as cancer-caused fatigue [6]. In addition, anxiety and depression in CRC survivors may continue to progress at higher levels over a longer period of time [7, 8]. Previous findings highlight the importance of reducing anxiety and depression in CRC survivors to continue treatment, improve fatigue and QOL.

Currently, effective interventions for psychological problems include both pharmacological and psychotherapeutic interventions. Psychotherapeutic interventions have fewer side effects than drugs, are more varied and flexible, and can meet the different needs of patients. It is increasingly being paid attention to by clinical workers and has become one of the recommended treatments in clinical guidelines [9]. Numerous studies have found that a variety of psychological interventions, such as cognitive intervention therapy [10, 11], mindfulness-based cognitive therapy [12], acceptance and commitment therapy [13], narrative therapy [14], and psychoeducational programmes [15], have a positive effect in alleviating negative emotions, fatigue and quality of life in cancer survivors. One evidence-based study confirmed that psychological interventions were associated with lower anxiety and depression scores and were able to improve the quality of life of CRC survivors [16]. However, previous studies have only conducted individual comparisons between psychological interventions, and there is a lack of head-to-head comparisons of different interventions with each other to clarify the effectiveness of each psychological intervention therapy on depression, anxiety, fatigue and quality of life improvement in CRC survivors.

Network Meta-analyses (NMA) can compare three or more interventions and can also rank interventions according to different outcomes, providing an evidence-based basis for clinical interventions [17]. Therefore, this study aims to make maximum use of the available evidence to answer the following independent review questions through systematic evaluations and network meta-analyses: ①To assess whether a psychological intervention improves anxiety and depression in CRC survivors compared with no treatment, sham treatment, or any other treatment (pairwise comparisons); ②To assess whether a psychological intervention improves physiological indicators of wellbeing in CRC survivors (fatigue and life quality of life); ③To rank the effectiveness of different types of psychological interventions (NMA).

## Methods

### Registration

This review was documented and registered in PROSPERO (No. CRD42023418397), a reputable platform for systematic reviews and meta-analyses. The research process will adhere to the guidelines established by the Preferred Reporting Items for Systematic Reviews and Meta-Analyses (PRISMA) extension statement for NMA and reporting systematic reviews [18]. Additionally, the current protocol will be reported using the Preferred Reporting Items for Systematic Review and Meta-Analysis Protocols checklist [19].

### Eligibility criteria

The criteria for inclusion and exclusion are established based on the patient, intervention, comparator, outcomes, and study design framework known as PICOS.

#### Participants (P)

This review focuses on patients diagnosed with colon cancer, rectal cancer, or combined colorectal cancer through gold standard pathological examination. The study population includes individuals aged 18 years or older, regardless of cancer stage, staging, surgical and radiotherapy modalities, and stoma status. Studies that do not report at least one primary or secondary outcome are excluded from this review.

#### Interventions (I)

This study includes psychological interventions by trained professionals in any setting or in any format (individual or group) and at any "dose" designed to improve depression, anxiety, fatigue, and quality of life for CRC survivors. Specific interventions include cognitive-behavioral interventions, mindfulness-based interventions, acceptance and commitment therapy, narrative therapy and psychoeducational programmes.

#### Comparators (C)

The control group chooses to receive routine nursing interventions or other kinds of interventions.

#### Outcomes (O)

The primary outcomes in this review are depression (e.g. Patient Health Questionnaire-9, PHQ-9) [20] and anxiety (e.g. Generalized Anxiety Disorder-7, GAD-7) [21]. Secondary outcomes are quality of life (e.g. Functional Assessment of Cancer Therapy–Colorectal, FACT-C) [22] and fatigue (e.g. Functional Assessment of Cancer Therapy-Fatigue subscale, FACT-fatigue) [23]. Without limiting the type of indicator assessment scales, change from baseline is measured preferably by validated assessment tools. According to the Cochrane handbook, we standardize the results of the different scales using standardized mean differences (SMDs) with 95% confidence intervals (CIs). The outcomes of this study are measured 6 months after the psychological intervention. If multiple assessments are conducted during this time interval, we assess the results closest to the end of the intervention.

#### Study design (S)

This review includes randomized controlled trials (RCTs) using psychological interventions to improve depression, anxiety, fatigue and quality of life in CRC survivors. The RCTs could be blinded, double blinded or unblinded and may have two or more arms.

### Search strategy and information sources

Research team members discuss together the appropriate databases and search terms to be searched. Considering that the languages of literature ultimately included in this review are Chinese and English, the finalized databases to be searched included PubMed, Embase, The Cochrane Library, Web of Science, CINAHL, PsycInFO, CNKI, and WanFang, with a timeframe of searching from the construction of the library to December 2023. The search terms are in the form of subject terms combined with free words, and a sensitive search filter to restrict randomized controlled trials. A draft search strategy of Pubmed is provided in Table 1. Detailed search strategies for other databases are in (Supporting Information–contains all the supporting tables).

**Table 1:** Search strategy of PubMed database.

Search	Query
#1	Search: (("Colorectal Neoplasms"[Mesh]) OR "Colonic Neoplasms"[Mesh]) OR "Rectal Neoplasms"[Mesh]
#2	Search: colorectal tumor*[Title/Abstract] OR colorectal cancer*[Title/Abstract] OR colorectal carcinoma*[Title/Abstract] OR colon adenocarcinoma*[Title/Abstract] OR colon* cancer*[Title/Abstract] OR cancer of the colon[Title/Abstract] OR rectal tumor*[Title/Abstract] OR rect* cancer*[Title/Abstract] OR rect* neoplasms[Title/Abstract] OR cancer of the rectum[Title/Abstract]
#3	#1 OR #2
#4	Search: ("Cognitive Behavioral Therapy"[Mesh]) OR "Cognitive Training"[Mesh] Sort by: Most Recent
#5	Search: cognitive behavioral therapy[Title/Abstract] OR CBT[Title/Abstract] OR cognitive therapy[Title/Abstract] OR cognitive behavi*[Title/Abstract] OR cognitive training[Title/Abstract] OR behavioral intervention[Title/Abstract] OR stress manage*[Title/Abstract] OR problem-sol*[Title/Abstract] OR problem adaptation therapy[Title/Abstract]
#6	Search: "Mindfulness"[Mesh] Sort by: Most Recent
#7	Search: "Meditation"[Mesh] Sort by: Most Recent
#8	Search: mindfulness[Title/Abstract] OR MBSR[Title/Abstract] OR MBCT[Title/Abstract] OR mindful*[Title/Abstract] OR relax*[Title/Abstract] OR yoga[Title/Abstract] OR meditation[Title/Abstract]
#9	Search: "Acceptance and Commitment Therapy"[Mesh] Sort by: Most Recent
#10	Search: "acceptance and commitment therapy"[Title/Abstract] OR ACT[Title/Abstract] OR acceptance therap*[Title/Abstract] OR commitment treatment*[Title/Abstract]
#11	Search: psychoeducational intervention[Title/Abstract] OR psychoeducational skill training[Title/Abstract] OR psychoeducat*[Title/Abstract]
#12	Search: "Narrative Therapy"[Mesh] Sort by: Most Recent
#13	Search: narrative therapy[Title/Abstract]
#14	#4 OR #5 OR #6 OR #7 OR #8 OR #9 OR #10 OR #11 OR #12 OR #13
#15	Search: "Randomized Controlled Trial" [Publication Type] Sort by: Most Recent
#16	Search: randomized controlled trial[Title/Abstract] OR randomized[Title/Abstract] OR randomly[Title/Abstract] OR RCT[Title/Abstract] OR RCTs[Title/Abstract]
#17	#15 OR #16
#18	#3 AND #14 AND #17

In addition to the above-mentioned electronic search, we conduct a manual search in references of included studies, grey literatures (clinical trial registries and preprints) and already published systematic reviews related to our topic. All studies are screened based on their titles and abstracts to examine their eligibility for inclusion.

### Study records

The search results are imported into Endnote X9 software and duplicate literatures are removed using the checking function. Two researchers trained in evidence-based nursing by Joanna Briggs Institute (JBI) independently screen the literatures according to the inclusion and exclusion criteria. Initial screening is performed after first reading the title and abstract, followed by re-screening by reading the full text to exclude non-compliant studies. In case of literatures with incomplete data information or data that could not be converted, attempts are made to contact the authors, and those that still could not be accessed were excluded to determine the final literature to be included. In case of disagreement, the decision is discussed by two people, and if consensus could not be reached by negotiation, it is referred to the third researcher for adjudication. Final study inclusion is presented in a PRISMA flow diagram.

### Data extraction

The two researchers utilize a standardized data extraction form, specifically designed for this study, to gather pertinent information from the literature. This form encompasses various elements such as the publication year, author, country, study population, sample size, details regarding the intervention and control groups, frequency of intervention, outcome indicators, as well as the tools used for measurement. Furthermore, in order to ensure comprehensive data collection, the researchers also extract specific data regarding each arm of the study. This includes information on the sample size, mean, and standard deviation for both the intervention and control conditions at various time points. These time points encompass the baseline (at the time of reporting) and post-treatment. Data extraction is completed by Excel 2016 software.

### Risk of bias in individual studies

The literature is evaluated using the Cochrane risk-of-bias tool (RoB 2.0) recommended for randomized controlled trials [24]. Five dimensions are evaluated: bias during randomization, bias in deviation from established interventions, bias in missing outcome data, bias in outcome measurement and bias in selective reporting of results. Each component is evaluated using the terms "low risk", " some concerns", and "high risk". An Excel file with macros accompanying the RCT evaluation tool is used as the evaluation software. Two JBI-trained review authors independently assess the risk of bias for each outcome. Any inconsistencies are resolved through discussion between the reviewers. In cases where consensus cannot be reached, a third party will be involved to make a final decision.

### Data synthesis

We conduct paired meta-analyses using a frequentist framework and a random effects model. The outcome indicators in this study are continuous variables, and effect values are expressed as standardized mean differences (SMD) and 95% confidence intervals (CI); when the 95% CI does not contain zero, a statistical difference between the two groups is considered to exist. The heterogeneity between head-to-head trials is estimated using I^2^ statistics and P-value. To assess transitivity, we construct a table of important clinical and methodological characteristics. Visual inspection is used to assess whether interventions are similar when included in randomized controlled trials of different designs and to compare the distribution of potential effect modifiers across pairwise comparisons.

Network Meta-analysis is performed using Stata 16.0 software calling the mvmeta package. We split the multi-arm studies in randomized controlled trials into two-arm studies for all combinations, and plot the network relationships of the different interventions, presenting direct versus indirect comparisons. Random effects frequency network meta-analyses are performed to assess differences in the effect of the examined interventions on primary and secondary outcomes after treatment.

The 95% CI and P-value for direct versus indirect comparisons of each psychological intervention are calculated using node-splitting analysis, the consistency of the direct and indirect comparisons of each split node is evaluated by the P-value. When P < 0.05 for each node comparison, it suggests that there is inconsistency in the network model, which is statistically analyzed using the inconsistency model and the reason for inconsistency is analyzed. When P > 0.05 when comparing across nodes, it suggests that there is no evidence of inconsistency in the network model, and the consistency model is used.

We rank the treatments using the P-score, which is a measure of the mean extent of certainty that a treatment is better than the competing treatments based on the frequentist approach [25]. The P-score ranges from 0 to 100%, and higher values indicate higher ranks. For meta-analyses involving at least 10 trials, we will explore potential and small study effects by generating funnel plots and evaluating them using linear regression tests [26]. We consider a P value of less than 0.1 significant for this test. In addition, we use comparison-adjusted funnel plots and the accompanying regression test to assess selection bias [27, 28].

When sufficient data are available, we conduct subgroup analyses or meta-regression analyses based on factors such as age of CRC survivors, cancer stage, duration of treatment or intervention, region of publication, and scales used to measure the outcomes. Subgroup analyses help us explore whether different regions and cultures have different understandings and measurements of anxiety, depression, fatigue, and quality of life, and whether these differences affect the evaluation of intervention effects. Scale analyses help us explore whether different measurement tools have an impact on the interpretation of the results. We also perform sensitivity analyses to explore the impact of variables on outcomes after excluding trials with high risk of bias or large amounts of missing data.

### Ethics and dissemination

No ethical approval is necessary as no individual participant data is being utilized. We anticipate presenting the results of the study to an academic conference and will publish them in a peer-reviewed journal.

## Discussion

As advances in cancer treatment have been made, the 5-year survival period for CRC has increased in tandem. The scientific management of physical and psychological symptoms such as anxiety, depression and fatigue experienced by cancer survivors to improve their quality of life during survival has become a priority for healthcare providers [4]. Previous studies have found that psychological interventions have a positive effect on improving physical and psychological problems in cancer survivors [29, 30]. However, there is still a lack of high-quality evidence reviews that summarize and compare the impact of different psychological interventions. To the best of our knowledge, this study is the first proposed qualitative and quantitative integration of existing evidence using systematic evaluation and network meta-analysis. The results of the study will inform health policy makers, healthcare providers’ clinical intervention choices and guideline revisions, and will help to reduce depression and anxiety in CRC survivors, reduce fatigue, improve quality of life, and promote disease recovery.

## Supporting information

S1 File Search strategy. (DOCX)

S1 Checklist PRISMA-P (Preferred Reporting Items for Systematic review and Meta-Analysis Protocols) 2015 checklist: Recommended items to address in a systematic review protocol*. (DOC)
