# Supplementary material for: Comparison of psychological interventions for anxiety, depression, fatigue and quality of life in colorectal cancer survivors: A systematic review and network meta-analysis protocol
Source: PLoS One. 2024 Apr 1;19(4):e0298589. doi: 10.1371/journal.pone.0298589 (PMC10984524; doi:10.1371/journal.pone.0298589)
Supplement: S1 File — (DOCX) [file pone.0298589.s001.docx]

**1. CNKI**

| **Search** | **Query** |
| --- | --- |
| #1 | (SU = '结直肠癌'+'结肠癌'+'直肠癌'+'结直肠肿瘤'+'结肠肿瘤'+'直肠肿瘤') AND ((SU = '认知行为疗法'+'认知疗法'+'认知干预'+'认知训练'+'认知心理疗法'+'压力管理'+'问题解决') OR (SU = '接纳与承诺疗法'+'接纳疗法'+'承诺疗法') OR (SU = '正念'+'放松'+'瑜伽'+'正性暗示'+'冥想') OR (SU = '心理教育'+'心理干预') OR (SU = '叙事疗法'+'叙事治疗'+'叙事护理')) |
| #2 | AB = '随机' |
| #3 | #1 and #2 |

**2. Wangfang**

| **Search** | **Query** |
| --- | --- |
| #1 | (主题：结直肠癌 or 结肠癌 or 直肠癌 or 结直肠肿瘤 or 结肠肿瘤 or 直肠肿瘤) and ((主题：认知行为疗法 or 认知疗法 or 认知干预 or 认知训练 or 认知心理疗法 or 压力管理 or 问题解决) or (主题：接纳与承诺疗法 or 接纳疗法 or 承诺疗法) or (主题：正念 or 放松 or 瑜伽 or 正性暗示 or 冥想) or (主题：心理教育 or 心理干预) or (主题：叙事疗法 or 叙事治疗 or 叙事护理)) |
| #2 | 主题：随机 |
| #3 | #1 and #2 |

**3. Web of Science**

| **Search** | **Query** |
| --- | --- |
| #1 | TS= (colorectal neoplasms OR colorectal tumor* OR colorectal cancer* OR colorectal carcinoma* OR colonic neoplasms OR colon adenocarcinoma* OR colon* cancer* OR cancer of the colon OR rectal tumor* OR rect* cancer* OR rect* neoplasms OR cancer of the rectum) |
| #2 | TS=（cognitive behavioral therapy OR CBT OR cognitive therapy OR cognitive behavi* OR cognitive training OR cognitive intervention* OR cognitive psychotherapy* OR stress manage* OR problem-sol* OR problem adaptation therapy OR behavioral intervention OR "acceptance and commitment therapy" OR ACT OR acceptance therap* OR commitment treatment* OR mindfulness OR MBSR OR MBCT OR mindful* OR relax* OR yoga OR meditation OR psychoeducational intervention OR psychoeducational skill training OR psychoeducat* OR psychotherapy OR narrative therapy） |
| #3 | TS=（randomized controlled trial OR randomized OR randomly OR RCT OR RCTs） |
| #4 | #1 AND #2 AND #3 |

**4. Embase**

| **Search** | **Query** |
| --- | --- |
| #1 | 'colorectal cancer'/exp OR 'colorectal cancer' OR 'colorectal tumor'/exp OR 'colorectal tumor' OR 'colon cancer'/exp OR 'colon cancer' OR 'colon tumor'/exp OR 'colon tumor' OR 'rectum tumor'/exp OR 'rectum tumor' OR 'rectum cancer'/exp OR 'rectum cancer' |
| #2 | 'colorectal neoplasms':ab,ti OR 'colorectal tumor*':ab,ti OR 'colorectal cancer*':ab,ti OR 'colorectal carcinoma*':ab,ti OR 'colonic neoplasms ':ab,ti OR 'colon adenocarcinoma*':ab,ti OR 'colon* cancer*':ab,ti OR 'cancer of the colon':ab,ti OR 'rectal tumor*':ab,ti OR 'rect* cancer*':ab,ti OR 'rect* neoplasm*':ab,ti OR 'cancer of the rectum':ab,ti |
| #3 | #1 OR #2 |
| #4 | 'cognitive behavioral therapy'/exp OR 'mindfulness'/exp OR 'mindfulness meditation'/exp OR 'psychotherapy'/exp OR 'meditation'/exp OR 'yoga'/exp OR 'acceptance and commitment therapy'/exp OR 'narrative therapy'/exp |
| #5 | 'cognitive behavioral therapy':ab,ti OR 'cognitive therapy':ab,ti OR 'cognitive psychotherapy*':ab,ti OR 'cognitive training':ab,ti OR 'behavioral intervention':ab,ti OR 'cognitive intervention*':ab,ti OR 'cbt':ab,ti OR 'stress manage*':ab,ti OR 'problem-sol*':ab,ti OR 'problem adaptation therapy':ab,ti OR 'mindfulness':ab,ti OR 'mbsr':ab,ti OR 'mbct':ab,ti OR 'mindful*':ab,ti OR 'meditation':ab,ti OR 'yoga':ab,ti OR 'acceptance and commitment therapy':ab,ti OR 'act':ab,ti OR 'acceptance therap*':ab,ti OR 'commitment treatment*':ab,ti OR 'narrative therapy':ab,ti OR 'psychoeducational intervention':ab,ti OR 'psychoeducational skill training':ab,ti OR 'psychoeducat*':ab,ti OR 'psychotherapy':ab,ti |
| #6 | #4 OR #5 |
| #7 | #3 AND #6 |
| #8 | **'randomized controlled trial'**/exp OR **'randomized controlled trial'**:ab,ti OR **'randomized'**:ab,ti OR **'randomly'**:ab,ti OR **'rct'**:ab,ti OR **'rcts'**:ab,ti |
| #9 | #7 AND #8 |

**5. The Cochrane library**

| **Search** | **Query** |
| --- | --- |
| #1 | MeSH descriptor: [Colorectal Neoplasms] explode all trees |
| #2 | MeSH descriptor: [Colonic Neoplasms] explode all trees |
| #3 | MeSH descriptor: [Rectal Neoplasms] explode all trees |
| #4 | (colorectal neoplasms):ti,ab,kw OR (colorectal tumor*):ti,ab,kw OR (colorectal cancer*):ti,ab,kw OR (colorectal carcinoma):ti,ab,kw OR (colonic neoplasms):ti,ab,kw OR (colon adenocarcinoma):ti,ab,kw OR (colon* cancer*):ti,ab,kw OR (cancer of the colon):ti,ab,kw OR (rectal tumor*):ti,ab,kw OR(rect* cancer*):ti,ab,kw OR (rect* neoplasm*):ti,ab,kw OR (cancer of the rectum):ti,ab,kw |
| #5 | #1 or #2 or #3 or #4 |
| #6 | MeSH descriptor: [Cognitive Behavioral Therapy] explode all trees |
| #7 | MeSH descriptor: [Cognitive Training] explode all trees |
| #8 | MeSH descriptor: [Mindfulness] explode all trees |
| #9 | MeSH descriptor: [Meditation] explode all trees |
| #10 | MeSH descriptor: [Yoga] explode all trees |
| #11 | MeSH descriptor: [Acceptance and Commitment Therapy] explode all trees |
| #12 | MeSH descriptor: [Narrative Therapy] explode all trees |
| #13 | (cognitive behavioral therapy):ti,ab,kw OR (cognitive therapy):ti,ab,kw OR (cognitive psychotherapy*):ti,ab,kw OR (cognitive training):ti,ab,kw OR (behavioral intervention):ti,ab,kw OR (cognitive intervention*):ti,ab,kw OR (cbt):ti,ab,kw OR (stress manage*):ti,ab,kw OR (problem-sol*):ti,ab,kw OR (problem adaptation therapy):ti,ab,kw OR (mindfulness):ti,ab,kw OR (mbsr):ti,ab,kw OR (mbct):ti,ab,kw OR (mindful*):ti,ab,kw OR (meditation):ti,ab,kw OR (yoga):ti,ab,kw OR (acceptance and commitment therapy):ti,ab,kw OR (act):ti,ab,kw OR (acceptance therap*):ti,ab,kw OR (commitment treatment*):ti,ab,kw OR (narrative therapy):ti,ab,kw OR (psychoeducational intervention):ti,ab,kw OR (psychoeducational skill training):ti,ab,kw OR (psychoeducat*):ti,ab,kw OR (psychotherapy):ti,ab,kw |
| #14 | #6 or #7 or #8 or #9 or #10 or #11 or #12 or #13 |
| #15 | #5 and #14 |
| #16 | MeSH descriptor: [Randomized Controlled Trial] explode all trees |
| #17 | (randomized controlled trial):ti,ab,kw OR (randomized):ti,ab,kw OR (Randomly):ti,ab,kw OR (RCT):ti,ab,kw OR (RCTs):ti,ab,kw |
| #18 | #16 or #17 |
| #19 | #15 and #18 |

**6. CINAHL**

| **Search** | **Query** |
| --- | --- |
| #1 | SU “colorectal neoplasms” OR “colorectal tumor*” OR “colorectal cancer*” OR “colorectal carcinoma” OR “colonic neoplasms” OR “colon adenocarcinoma” OR “colon* cancer*” OR “cancer of the colon” OR “rectal tumor*” OR “rect* cancer*” OR “rect* neoplasm*” OR “cancer of the rectum” |
| #2 | SU “cognitive behavioral therapy” OR “cognitive therapy” OR cognitive psychotherapy* OR cognitive training OR behavioral intervention OR cognitive intervention* OR CBT OR stress manage* OR problem-sol* OR “problem adaptation therapy” OR mindfulness OR MBSR OR MBCT OR mindful* OR meditation OR yoga OR “acceptance and commitment therapy” OR ACT OR “acceptance therap*” OR commitment treatment* OR “narrative therapy” OR psychoeducational intervention OR psychoeducational skill training OR psychoeducat* OR psychotherapy |
| #3 | #1 and #2 |
| #4 | randomized controlled trial OR randomized OR randomly OR RCT OR RCTs |
| #5 | #3 and #4 |

**7. PsycINFO**

| **Search** | **Query** |
| --- | --- |
| #1 | SU colorectal neoplasms OR colorectal tumor* OR colorectal cancer* OR colorectal carcinoma OR colonic neoplasms OR colon adenocarcinoma OR colon* cancer* OR cancer of the colon OR rectal tumor* OR rect* cancer* OR rect* neoplasm* OR “cancer of the rectum” |
| #2 | SU “cognitive behavioral therapy” OR “cognitive therapy” OR cognitive psychotherapy* OR cognitive training OR behavioral intervention OR cognitive intervention* OR CBT OR stress manage* OR problem-sol* OR “problem adaptation therapy” OR mindfulness OR MBSR OR MBCT OR mindful* OR meditation OR yoga OR “acceptance and commitment therapy” OR ACT OR “acceptance therap*” OR commitment treatment* OR “narrative therapy” OR psychoeducational intervention OR psychoeducational skill training OR psychoeducat* OR psychotherapy |
| #3 | #1 and #2 |
| #4 | randomized controlled trial OR randomized OR randomly OR RCT OR RCTs |
| #5 | #3 and #4 |
